# Inducing hierarchical pores in nano-MOFs for efficient gas separation†

**DOI:** 10.1039/d3ra01175e

**Published:** 2023-05-30

**Authors:** Kritika Narang Landström, Ashwin Nambi, Andreas Kaiser, Farid Akhtar

**Affiliations:** a Division of Materials Science, Luleå University of Technology Luleå 97187 Sweden farid.akhtar@ltu.se; b Department of Energy Conversion & Storage, Technical University of Denmark 2800 Kgs Lyngby Denmark

## Abstract

The synthesis of metal–organic frameworks (MOFs) and their processing into structures with tailored hierarchical porosity is essential for using MOFs in the adsorption-driven gas separation process. We report the synthesis of modified Cu-MOF nanocrystals for CO_2_ separation from CH_4_ and N_2_, prepared from DABCO (1,4-diazabicyclo[2.2.2] octane) and 9,10 anthracene dicarboxylic acid linkers with copper metal salt. The synthesis parameters were optimized to introduce mesoporosity in the microporous Cu-MOF crystals. The volumetric CO_2_ adsorption capacity of the new hierarchical Cu-MOF was 2.58 mmol g^−1^ at 293 K and 100 kPa with a low isosteric heat of adsorption of 28 kJ mol^−1^. The hierarchical Cu-MOF nanocrystals were structured into mechanically stable pellets with a diametral compression strength exceeding 1.2 MPa using polyvinyl alcohol (PVA) as a binder. The CO_2_ breakthrough curves were measured from a binary CO_2_–CH_4_ (45/55 vol%) gas mixture at 293 K and 400 kPa pressure on Cu-MOF pellets to demonstrate the role of hierarchical porosity in mass transfer kinetics during adsorption. The structured hierarchical Cu-MOF pellets showed stable cyclic CO_2_ adsorption capacity during 5 adsorption–desorption cycles with a CO_2_ uptake capacity of 3.1 mmol g^−1^ at 400 kPa and showed a high mass transfer coefficient of 1.8 m s^−1^ as compared to the benchmark zeolite NaX commercialized binderless granules, suggesting that the introduction of hierarchical porosity in Cu-MOF pellets can effectively reduce the time for CO_2_ separation cycles.

## Abstract 2

Structured hierarchically porous pellets of Cu-MOF nanocrystals show interesting properties for CO_2_ capture.

## Graphical abstract

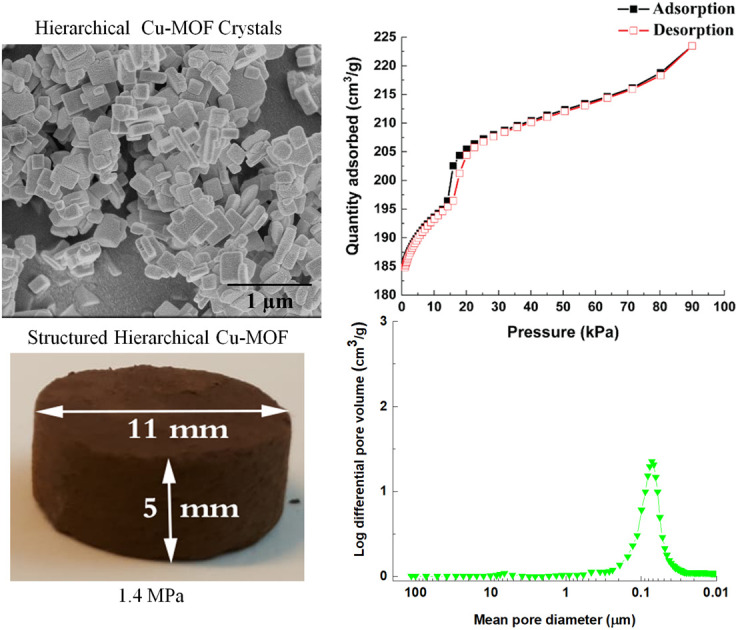

## 1 Introduction

The porosity and adsorptive properties of adsorbents have been essential to design novel materials for adsorption-driven gas separation technologies.^1,2^ Amongst adsorbents, metal–organic frameworks (MOFs) are a promising class of porous materials with a 3D crystalline structure and offer unique intrinsic characteristics such as ultrahigh surface area, open pore channels, functional tunability, and versatile geometry, implying their extensive applications in gas separation and energy storage technology.^3–5^ With these inherent structural and chemical characteristics, extensive research has been focused on developing high-performance MOFs for CO_2_ separation from gas streams.^4–6^ Experimental results have shown that MOFs such as Mg-MOF-74, UIO-66(Zr)-(OH)_2_, and SIFSIX-2-Cu-i have outperformed the benchmark zeolites NaX and CaA with remarkable CO_2_ adsorption capacities of 8.2 mmol g^−1^, 5.6 mmol g^−1^, and 5.4 mmol g^−1^ at 1 bar and ambient temperature.^7–9^ Compared to traditional adsorbents such as zeolites, silica, and activated carbon, MOFs can be precisely augmented for selective CO_2_ adsorption by creating open metal sites and Lewis basic sites to enhance CO_2_ binding affinities.^10^ For instance, IRMOF-3 (isorecticular to MOF-5) utilizes an amine functionalized organic linker to exhibit a higher CO_2_ adsorption capacity 14.7 mmol g^−1^ at 12.3 bar, 298 K as compared to MOF-5, 14.0 mmol g^−1^.^11^ Similarly, amine-functionalized MIL-100 and MIL-101 have enhanced CO_2_/CH_4_ selectivity with CO_2_ adsorption capacities of 3.5 mmol g^−1^ and 6.1 mmol g^−1^ at 10 bar and 298 K, respectively.^12^ G. E. Cmarik and co-workers conducted a comparative study on amino, nitro, and methoxy-functionalized UIO-66 MOF to obtain basic sites for high CO_2_ selectivity. They showed that amine-functionalized UIO-66 exhibits high CO_2_/N_2_ and CO_2_/CH_4_ selectivity of 61.5 and 18.3, respectively.^7^ Apart from amine functionalization, other nucleophilic linkers such as azoles, pyrimidines, and triazines could be used to obtain Lewis basic sites. Lv *et al.* developed a MOF with imidazole group and displayed high CO_2_/N_2_ and CO_2_/CH_4_ selectivity of 252 and 151, respectively.^13^ Similarly, in this study, we have incorporated 1,4-diaazbicyclo [2.2.2] octane (DABCO), a nitrogen containing nucleophilic linker with a tertiary amine group, in the synthesis Cu-MOFs to achieve high CO_2_ adsorption capacities over N_2_ and CH_4_. Furthermore, the excess ligands and/or modulators and synthesis in dilute conditions lead to the synthesis of nano-MOFs.^14^

Despite a decade of extensive research on MOFs adsorbents, their application in industrial CO_2_ separation is limited due to a lack of effective structuring at multivariate length scales. Further studies in gas separation have shown that the structuring of adsorbents at the nanoscale lead to promising adsorption capacity and selectivity due to larger surface area and more rapid adsorption kinetics compared to the traditional use of large MOF crystals.^15–17^ Hence, large efforts have been recently devoted to synthesizing and structuring nanocrystallites into hierarchical porous MOF for use as adsorbents.^18,19^ X. Mu *et al.* developed a nanoscale HKUST-1 MOF with a high specific BET surface area of 1542.4 m^2^ g^−1^ and a CO_2_ uptake capacity of 2.5 mmol g^−1^.^20^ In this context, numerous efforts have been made to design facile methods such as microwave, mechanochemical, and ultrasound synthesis to develop MOF nanocrystals.^21–24^ It is important to note that synthesis must be optimized to achieve uniform particle size distribution and well-defined morphology by controlling the nucleation and growth rate. However, tailoring of CO_2_ adsorptive properties for MOF nanocrystals by introducing hierarchical porosity and structuring in separation technology can potentially develop robust materials for practical applications.

Numerous gas separation technologies, such as pressure swing adsorption (PSA), vacuum swing adsorption (VSA), temperature swing adsorption (TSA), and membrane-based technology,^25,26^ are being used to investigate the performance of adsorbents for the separation of different gases. PSA technology is one of the preferred technologies in industrial biogas upgrading plants due to high efficiency (95–98%), low energy consumption, and low maintenance cost.^27,28^ To date, MOFs are usually synthesized in the form of particles/powders, which are disadvantageous to use in PSA columns due to high-pressure drop across the column and high attrition rate, which can lead to the fluidization of the adsorption bed at high pressures and result in slower mass and heat transfer kinetics. To address these issues, the MOF powders should first be structured into hierarchical pellets, extrudates or granules to impart high mechanical strength, better heat transfer management, and facilitate high mass transfer kinetics by enhancing the molecular accessibility to the adsorption sites through multiscale pore regime. Recently, advanced nanostructuring of zeolite and MOF adsorbents into hierarchical porous nanofibers has been investigated for energy and environmental applications, including biogas separation and storage.^29–32^ Zhang *et al.* fabricated hierarchical porous ZSM-5 nanocomposite pellets for CO_2_ separation from a biogas stream, which revealed a 30% increase in specific BET surface area, a 34% increase in CO_2_ adsorption capacity as compared to ZSM-5 nanopowder. Apart from the high CO_2_ uptake capacity, these structured pellets with 46% porosity displayed a high mass transfer coefficient of 1.24 m s^−1^ in the adsorption column.^29^ Similarly, in our earlier work, K. Narang *et al.* structured hierarchical NaX and CaA zeolite granules and investigated the potential for CO_2_ separation performance in a PSA unit. The hierarchical NaX and CaA zeolite granules showed enhanced mass transfer coefficients of 1.3 m s^−1^ and 1.6 m s^−1^ in the adsorption column as compared to previously reported structured zeolites.^33^ From the previously reported studies on structured zeolites NaX, CaA, and activated carbon, it is established that the efficiency of PSA technology is influenced by the adsorbent characteristics. However, the assessment of hierarchically structured nano-MOFs for CO_2_ separation is relatively sparse, and therefore, the potential of these materials in gas separation applications is still to be demonstrated.

Here, we describe a method for the synthesis and structuring of a nanocrystalline MOF, using copper as a metal centre and DABCO and H_2_ADC as organic linkers, named Cu-MOF. We report the assembly of the MOF nanocrystallites into a hierarchical porous structure (mechanical stable pellets) with additional meso- and macropores and report the textural characteristics, the CO_2_ adsorption capacity, and the calculation of mass transfer kinetics from breakthrough curves. The present work elucidates the advantages of structured hierarchically porous Cu-MOFs, and the results can be extended to other MOF materials.

## 2 Experimental section

### 2.1 Materials

Copper(ii) nitrate hemi (pentahydrate) [Cu(NO_3_)_2_·2.5H_2_O] (≥99.99%), *N*,*N*-dimethylformamide (DMF), 1,4-diazabicylco[2.2.2] octane [DABCO] (≥99%), 9,10-anthracenedicarboxylic acid [H_2_ADC] (95%) were purchased from Sigma Aldrich Chemie GmBH, Germany. All the above-mentioned chemicals were used as received.

### 2.2 Synthesis of Cu-MOF and hierarchical Cu-MOF

For the synthesis of the Cu-MOF, a metal salt solution was prepared by dissolving Cu(NO_3_)_2_·2.5H_2_O (233 mg, 1 mmol) to DMF (9 ml). A mixture of DABCO (93.1 mg, 0.83 mmol) and H_2_ADC (266.25 mg, 1 mmol) were prepared in DMF (18 ml) and stirred using a magnetic stirrer until dissolved. Further, the above prepared organic linker solution was added to the metal salt solution and stirred for 20 min. The reaction mixture was transferred to a 30 ml autoclave and heated at an elevated temperature of 120 and 150 °C for 48 h to yield a brown color crystalline material. Several synthesis parameters (amount of solvent, temperature, reaction temperature, and metal-linker ratio) were optimized in the synthesis of the Cu-MOF to achieve additional mesoporosity in the MOF. These parameters are listed in the ESI, Table S1.†

**Fig. 1:**
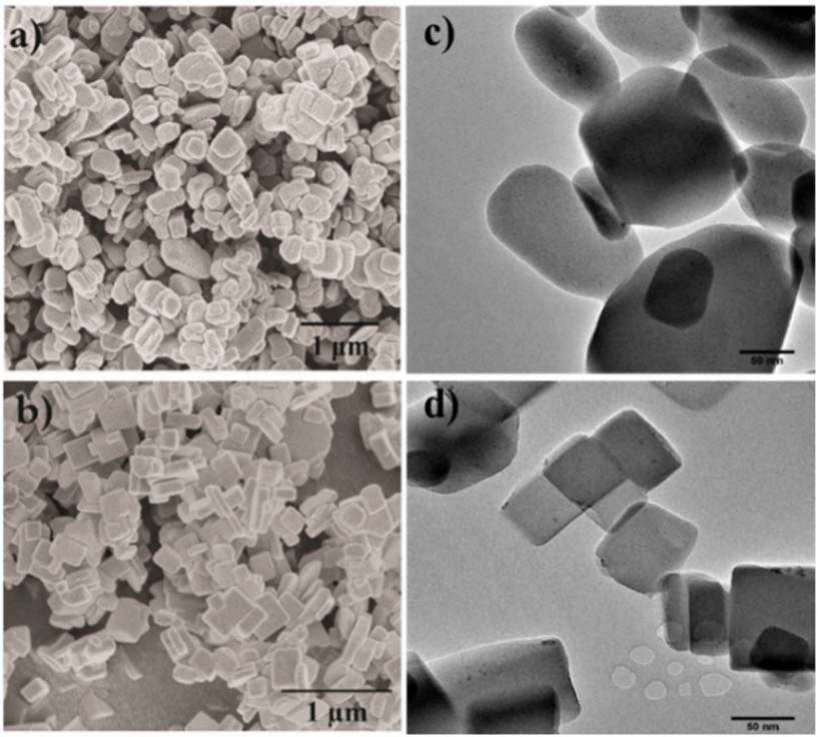
SEM micrographs and TEM images of Cu-MOF (composition A (a) and (c)) and hierarchical Cu-MOF (composition E in (b) and (d)).

For the synthesis of the hierarchical Cu-MOF, the metal salt and organic linker solution were prepared in the same manner as described in the Cu-MOF synthesis above. The molar ratio of metal salt to organic linkers remained the same, whereas the solvent amount was increased to at least 70 ml. Further, the solution was transferred into a 100 ml autoclave and heated at 120 °C for 48 h. The MOF composition with the highest specific BET surface area (composition E) was selected for further investigation and called hierarchical porous MOF (Table 1S, and Fig. 1S†).

The solvent exchange was performed on the MOF compositions. For this purpose, the synthesized MOFs were washed with pure DMF to remove the excess unreacted constituents. Additionally, the synthesized MOF crystals were washed 3 times per day with ethanol for three days to prevent the framework from collapsing during the thermal activation under vacuum.

### 2.3 Pelletization

The nanocrystalline MOF powders were structured into pellets of 11 mm in diameter, using a warm compaction process that was carried out at 100 °C and 20 MPa pressure. Before the structuring, the MOF nanocrystals were dry mixed thoroughly with the 20 wt% polyvinyl alcohol (PVA) binder to impart mechanical strength.

### 2.4 Characterization

Microstructure and morphology were analyzed using FEI Magellan 400 field emission XHR-SEM. Prior to the analysis; the samples were coated with a 30 nm layer of platinum to avoid the accumulation of charges, using Leica EMACE 200 coating equipment. The particle size of the sample was measured using Adobe Photoshop CC 2019 software. Whereas, the MOF's structural analysis was conducted using a PANalytical Empyrean powder X-ray diffractometer (Malvern, UK) equipped with PIXcel 3D detector. Monochromatic Cu Kα radiation (*λ* = 0.154 nm) was used as an X-ray source and operated at 40 kV and 45 mA with a scan rate of 0.02°. The thermal stability of MOF was evaluated using simultaneous thermal analysis with an STA 449 F3 from Netzsch, Germany (Fig. 1S†).

### 2.5 Adsorptive studies

Single CO_2_ and CH_4_ adsorption measurements were conducted using a Gemini VII 2390 Surface Area Analyzer (Micrometrics, Norcross, USA) within a pressure range of 1 to 90 kPa to study the microscopic sorption characteristics of the MOFs. The isotherm data obtained from the N_2_ adsorption experiment at −196 °C (77 K) were used to determine the specific BET (Brunauer–Emmett–Teller) surface area and other textural properties of the MOFs. Additional measurement points at low pressure were added for the N_2_-adsorption–desorption measurements at −196 °C (77 K) to improve the reliability of data. Prior to the analysis, the samples were regenerated at 190 °C using dynamic vacuum VacPrep 061 for 12 h to eliminate all the pre-adsorbed species. The CO_2_-over-CH_4_/N_2_ selectivities of the MOFs were calculated using Henry's law. The isosteric heat of adsorption was calculated using Clausius–Clapeyron equation on adsorption equilibrium data obtained at three different temperatures of 30 °C, 0 °C, and −30 °C. Mercury intrusion porosimetry was performed on the Cu-MOF and the hierarchical Cu-MOF pellets and powders. For this purpose, a Micrometrics AutoPore III-9400 (Norcross, GA, USA) was used over a wide pressure range of 0.1–400 MPa. The pore size distributions were evaluated using the Washburn equation with a surface tension and contact angle of mercury of 0.485 N m^−1^ and 130°, respectively. The gravimetric CO_2_, N_2,_ and CH_4_ isotherms were obtained from an ISOSORP Sorption Analyser (TA instruments, Germany) using a magnetic suspension balance (MSB). Prior to the measurement, the samples were pre-treated at 190 °C for 6 h. Furthermore, the samples undergo a buoyancy experiment using helium gas to determine the fluid density in a buoyancy correction before the start of the adsorption measurements. All the adsorption isotherms were carried out at 22 °C up to 10 bars.

Five adsorption–desorption cyclic breakthrough experiments were performed on a lab-scale fixed-dual beds Pressure Swing Adsorption unit (PSA-300 LC, LC Science and Technology, Florida, USA) to investigate the CO_2_ separation performance on the structured pellets. The PSA bed (length = 100 mm and diameter = 15 mm) was filled with structured pellets. A gas stream of CH_4_/CO_2_ (55/45 vol%) was fed into the column with a volumetric flow of 75 ml min^−1^ and 60 ml min^−1^, at 4 bars and 20 °C. Before the first cycle, the pellets were activated at 190 °C under a helium flow 20 ml min^−1^ for 12 h and the subsequent cycles, the adsorbed gases were evacuated using dynamic vacuum. A dew point of −22 °C was considered a standard to ensure efficient drying. The Cu-MOF pellets and hierarchical Cu-MOF pellets loaded in the PSA bed were 2.8 g at a column height of 27 mm and a loading of 2.1 g at a column height of 17 mm, respectively. These breakthrough experiments were used to calculate the mass transfer kinetics of the gases through the packed bed structure of the prepared MOFs.

## 3 Results and discussion

The morphology and particle size of the Cu-MOFs from the different synthesis routes were characterized by scanning electron microscopy (SEM) and transmission electron microscopy (TEM). Fig. 1a and b show the morphology and particle size of the Cu-MOF nanocrystals and the hierarchical Cu-MOF nanocrystals by SEM micrographs to illustrate the influence of the hierarchical porous network on the CO_2_ adsorption and separation from a CH_4_ and N_2_ mixture. The prepared pristine Cu-MOF and hierarchical Cu-MOF nanocrystals exhibit cuboid morphology in a size range of 150–650 nm and 150–450 nm, respectively. The results show that the growth rate of the Cu-MOF crystals could be reduced in the solvothermal synthesis process by tailoring the molar ratio of the organic linkers and the metal salt. An excessive amount of organic linker in the solution can slow the growth rate of MOF nanocrystals by surmounting the diffusion of metal ions.^14^Fig. 1c and d show the TEM images with small primary crystallites with rounded edges for the Cu-MOF, whereas the hierarchical Cu-MOF reveals crystallites with sharper (less rounded) edges.

**Fig. 2:**
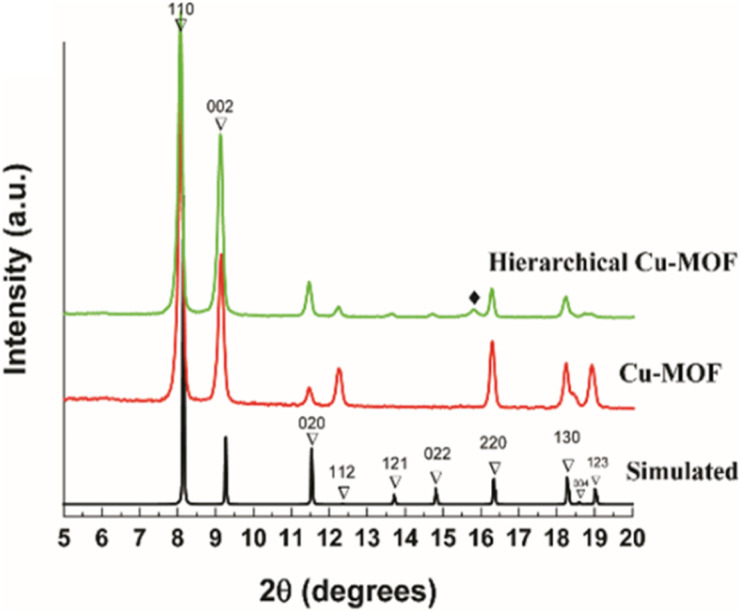
PXRD patterns (bottom) of Cu-MOF (red), hierarchical Cu-MOF (green) and the simulated pattern of Zn_2_(adc)_2_(dabco) MOF (black).^35^

**Fig. 3:**
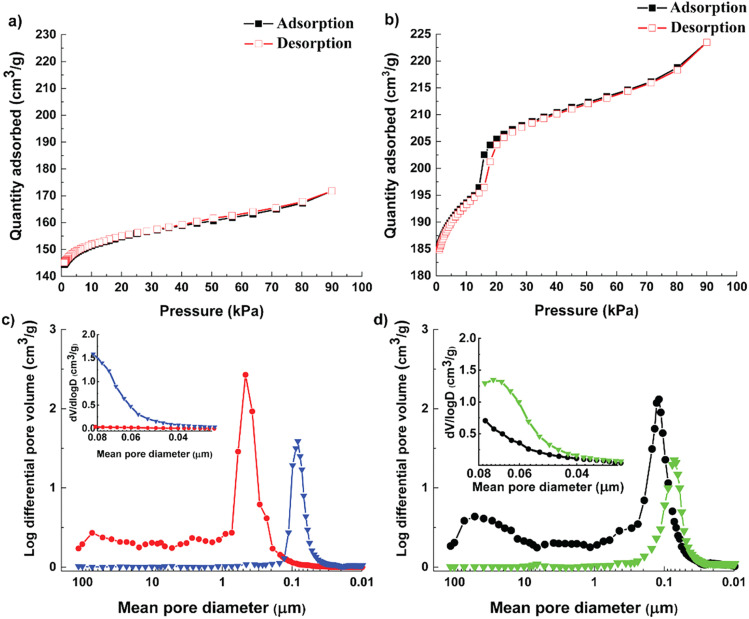
N_2_ adsorption (black, square) and desorption (red, hollow square) isotherms collected at −196 °C for (a) Cu-MOF nanocrystals, (b) hierarchical Cu-MOF nanocrystals; pore size distribution obtained from mercury intrusion porosimetry with the inset covering mean pore diameters below 0.08 μm for (c) Cu-MOF powder (red, circle) and Cu-MOF pellet (blue, inverted triangle) and (d) hierarchical Cu-MOF powder (black, circle) and pellet (green, inverted triangle).

The powder X-ray diffractograms (PXRD) were obtained for Cu-MOF and hierarchical Cu-MOF nanocrystals and are illustrated in Fig. 2. The obtained PXRD were compared with the simulated pattern of the analogous activated Zn_2_(adc)_2_(dabco) MOF procured from the crystallographic database (Fig. 3S†).^34^ The PXRD shows that the synthesized Cu-MOF and hierarchical Cu-MOF were confirmed to be isostructural to activated Zn_2_(adc)_2_(dabco) as established from the matched XRD reflections. Both the synthesized MOF nanocrystals crystallize in tetragonal space group *I*4/*mcm*. Based on Bragg's peak positions, the calculated unit cell parameters of the hierarchical Cu-MOF are *a* ∼ 15.29 Å, *b* ∼ 15.29 Å, and *c* ∼ 19.15 Å in the crystallographic cell. The minor discrepancies, such as the absence of peaks 121, 022 and the presence of peak (marked with ♦), may be attributed to the structural changes caused by guest molecules.^35^ Thermal stability results obtained from TGA analysis (Fig. 2S†) indicate better thermal stability of hierarchical Cu-MOF than the pristine Cu-MOF.

Textural properties and pore size distribution of the Cu-MOF and the hierarchical Cu-MOF were investigated by N_2_ adsorption–desorption isotherms (Fig. 3, and Table 1). The as-synthesized Cu-MOF showed Type I isotherm with high uptake in the low-pressure regime, signifying the presence of micropores. Additionally, the Cu-MOF showed a narrow pore size distribution with a microporous region with a maximum at 11.4 Å, calculated from the N_2_-adsorption–desorption isotherm collected at −196 °C using the DFT pore size model in the Gemini VII 2370 analysis software, resulting in a similar pore size to ZIF-8 and a benchmark MOF-5.^5^

**Table 1:** Textural properties, Henry's selectivity and isosteric heat of adsorption of Cu-MOF and hierarchical Cu-MOF. Textural properties were obtained from N_2_ adsorption measurements at 196 °C, Henry's selectivity was obtained from and CO_2_ and CH_4_ adsorption isotherms recorded at 20 °C, and isosteric heat of adsorption was obtained from CO_2_ adsorption isotherms recorded at 30 °C, 0 °C, and −30 °C

MOF	BET surface area (m^2^ g^−1^)	Micropore area (m^2^ g^−1^)	External surface area (m^2^ g^−1^)	Micropore volume (cm^3^ g^−1^)	Pore size (Å)	Henry selectivity CO_2_/N_2_ (CO_2_/CH_4_)	Isosteric heat of adsorption Δ*H*_CO_2__ (kJ mol^−1^)
Cu-MOF	444.4	373	71.4	0.19	11.6	9 (2.4)	48
Hierarchical Cu-MOF	627.4	444.3	201.4	0.22	27.4	12 (2.9)	28

However, the hierarchical Cu-MOF also exhibits a Type IV isotherm (Fig. 3b) according to the IUPAC classification with a H1-type hysteresis around 20 kPa, which is a typical feature of mesoporous structures. The hierarchical Cu-MOF also showed sharp uptake at low-pressure regime, signifying the co-existence of micro–meso porosity. Similarly, Jiang *et al.* synthesized MIL-101 possessing mesopores of 29 Å and 34 Å to facilitate CO_2_ conversion.^36^Fig. 3c show the pore size distribution obtained from mercury intrusion porosimetry before and after pelletization for the Cu-MOF and Fig. 3d for the hierarchical Cu-MOF. The structured hierarchical Cu-MOF displayed a mean pore diameter at around 110 nm, corresponding to the inter-particle distance between the nanocrystals. This inter-particle distance is larger for the Cu-MOF powder (peak around 450 nm) than the hierarchical Cu-MOF due to relatively larger nanocrystals. Thus, the reduction of nanocrystallite size of Cu-MOF powder and the assembly into a hierarchically porous structure (pelletizing) led to a significant increase of mesopore volume with reduced mean pore diameter (smaller pore channels, see Fig. 3d) in the resulting hierarchical porous Cu-MOF. This observation agrees with the SEM images in Fig. 1. A similar difference in the pore size can be observed for the hierarchical and pristine Cu-MOF structured pellets at lower mean pore diameter of 70 and 80 nm, respectively.

The textural characteristics of the synthesized MOFs are displayed in Table 1. The hierarchical Cu MOF has 41% increase in the BET surface area to 627 m^2^ g^−1^ with a micropore volume of 0.22 cm^3^ g^−1^. A significant increase of 182% in the external surface is noticed for the hierarchical Cu-MOF. The Cu-MOF powder showed a high isosteric heat of adsorption (ΔHCO_2_) of 48 kJ mol^−1^, which could be expected due to the presence of the nucleophilic organic linker DABCO. The heat of adsorption for Cu-MOF was slightly higher than the benchmark Mg-MOF-74 (47 kJ mol^−1^), which signifies a strong affinity of the framework for CO_2_ molecule. In contrast, for the hierarchical Cu-MOF, the heat of adsorption (ΔHCO_2_) was found to be significantly lower with only 28 kJ mol^−1^, which implies a lower energy demand required for regeneration. In the Cu-MOF, the CO_2_ molecules are mostly confined in narrow micropores, which increases the interaction between them and results in high heat of adsorption. On the other hand, in hierarchical Cu-MOF, the CO_2_ molecules are adsorbed in larger pores including a larger meso pore volume with small mesopores of 27 Å (determined applying the DFT pore size model in the Gemini VII 2390 software), resulting in an overall increased external surface area. Hence, CO_2_ molecules possess fewer interactions between them due to more accessible space while filling up the pores. The results of our study are in good agreement with a study on hierarchical porous carbon (HPC), in which a HPC material with large fraction of mesopores showed a lower heat of adsorption compared to HPC with low fraction of mesopores.^37^

**Fig. 4:**
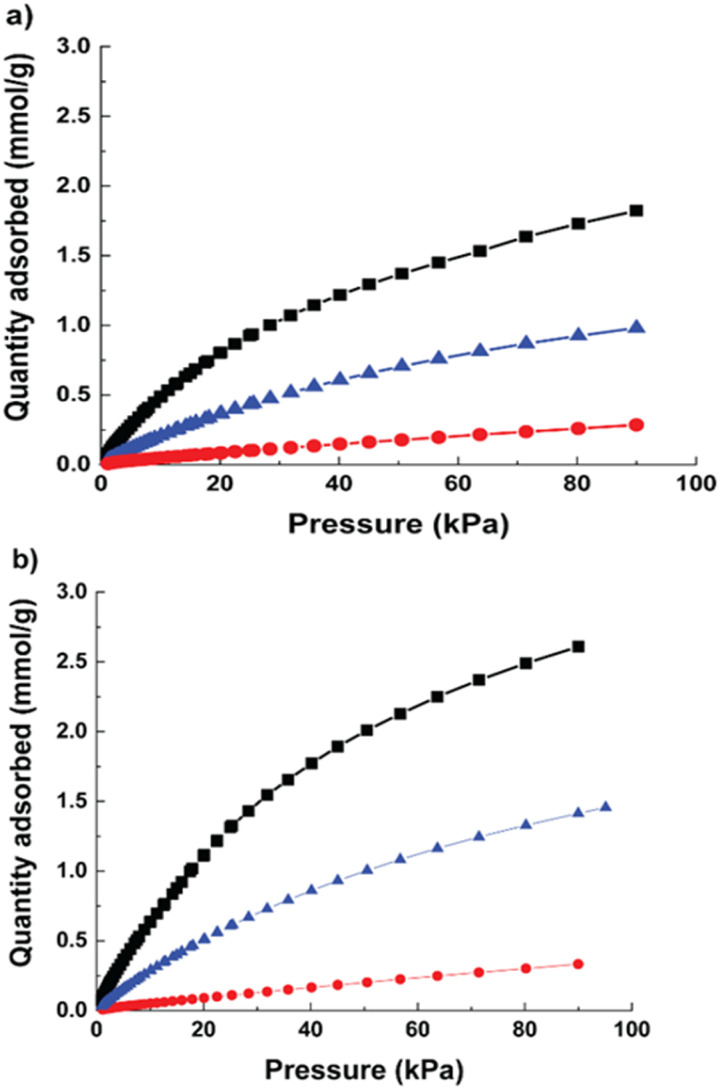
Adsorption isotherms at 293 K (a) Cu-MOF (b) hierarchical micro–mesoporous Cu-MOF; CO_2_ (black, square); CH_4_ (blue, triangle); N_2_ (red, circle).

The CO_2_, CH_4_ and N_2_ uptake of Cu-MOF and hierarchical Cu-MOF are shown in Fig. 4 at 293 K. Both MOFs showed type 1 CO_2_ adsorption isotherm caused by the presence of strong interaction between the CO_2_ quadruple moment (−14 × 10^−40^ C m^2^) and the adsorbent. The hierarchical Cu-MOF has shown adsorption of 2.58 mmol g^−1^ CO_2_ at 1 bar, which is 46% higher CO_2_ uptake compared to the Cu-MOF. This is due to the presence of additional mesopores and the corresponding enhanced BET surface area. The significant increase in the CO_2_ equilibrium capacity in combination with a relative low heat of adsorption makes it a promising candidate for efficient CO_2_ separation. The CH_4_ adsorption capacity for Cu-MOF and hierarchical Cu-MOF was 1 mmol g^−1^ and 1.4 mmol g^−1^, respectively. N_2_ adsorption uptake was significantly lower in both MOF nanocrystals due to the much lower quadruple moment of N_2_ (−4.6 × 10^−40^ C m^2^). Furthermore, the single gas adsorption isotherms were used to investigate CO_2_ separation performance from CH_4_ and N_2_ by estimating Henry's selectivity, and the results are given in Table 1. Here, the hierarchical Cu-MOF showed a 33% increase in CO_2_-over-N_2_ selectivity, whereas no significant difference in CO_2_-over-CH_4_ selectivity was observed compared to the Cu-MOF.

**Fig. 5:**
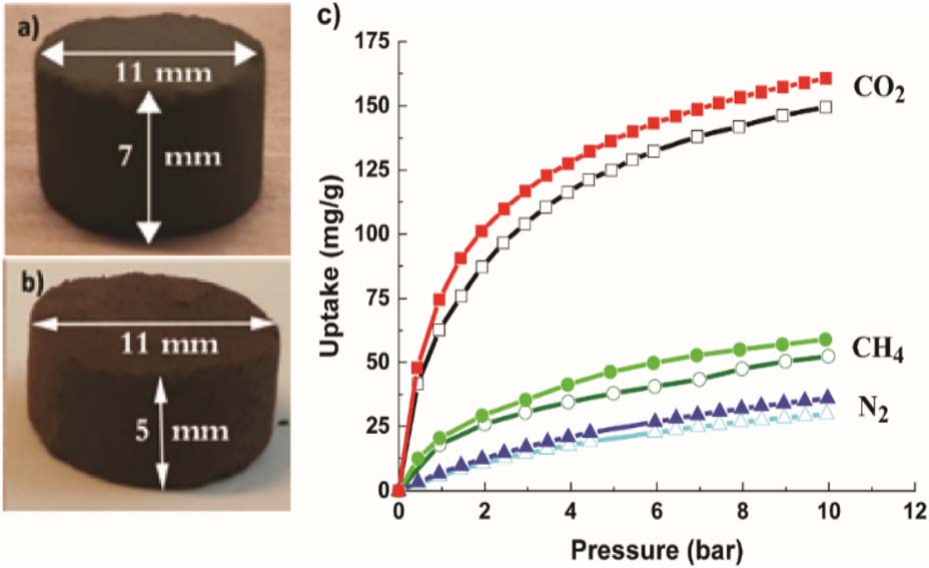
Image of (a) Cu-MOF and (b) hierarchical Cu-MOF; (c) gravimetric adsorption isotherms up to 10 bar for Cu-MOF (hollow symbols) and hierarchical Cu-MOF (filled symbols). CO_2_ (red, square); CH_4_ (green, circle) and N_2_ (blue, triangle).

For breakthrough experiments, the Cu-MOF and the hierarchical Cu-MOF nanocrystals were structured into pellets to impart mechanical strength to withstand high pressure and fluctuations in gas flow in a packed bed. The average diametrical compression strength was found to be 1.2 MPa for Cu-MOF and 1.4 MPa for hierarchical Cu-MOF. In fact, both pellets were intact after high-pressure gas uptake (up to 10 bar) in gravimetric adsorption experiments in N_2_, CO_2_ and CH_4_, as shown in Fig. 5. The hierarchical Cu-MOF pellet showed 7% higher gravimetric CO_2_ uptake capacity, 160 mg g^−1^ than Cu-MOF (148 mg g^−1^) owing to a larger pore volume. The results show that the structured pellets retained the adsorptive properties even with the presence of binder. The gravimetric CO_2_ adsorption uptake of the hierarchical Cu-MOF was higher than the uptake of an activated carbon/TEA (triethanolamine) with 158 mg g^−1^ and similar to NaX/MEA (monoethanolamine) composite with 162 mg g^−1^ at 298 K and 10 bar.^38^ Table 2S† summarizes the values for selectivity and CO_2_ uptake for various MOFs reported in literature.

**Fig. 6:**
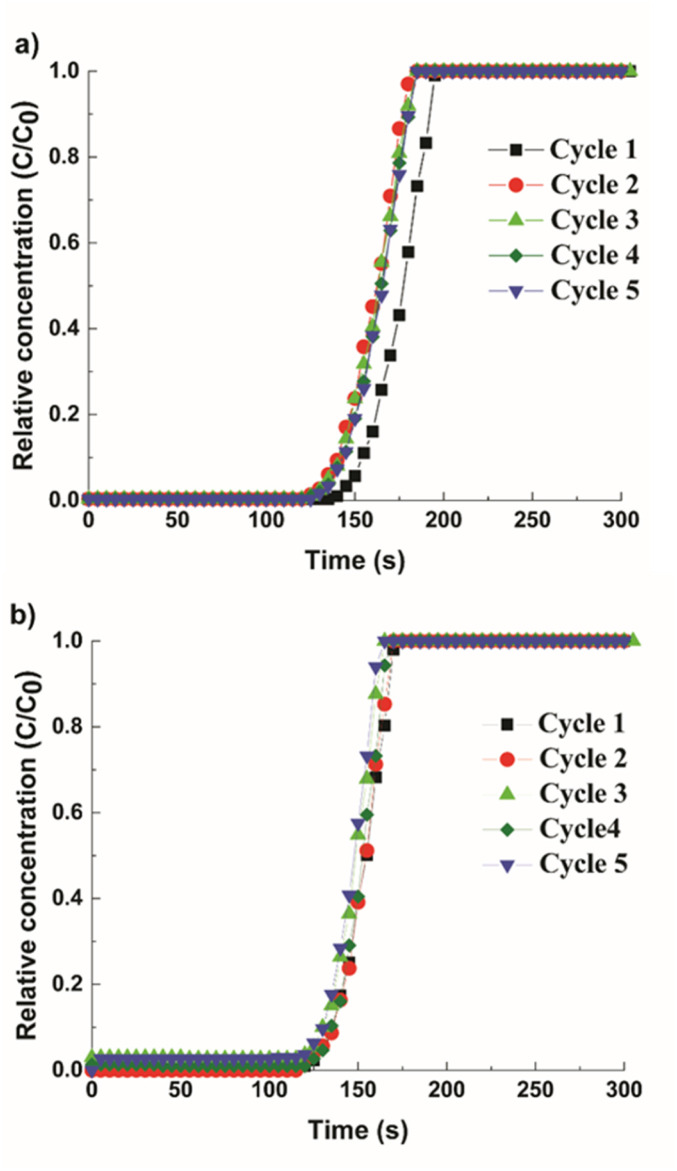
Breakthrough curves with a mixture of CH_4_/CO_2_ from cycle 1 to cycle 5. (a) Cu-MOF pellets and (b) hierarchical Cu-MOF pellets. Cycle 1 (black, square); cycle 2 (red, circle); cycle 3 (green, triangle); cycle 4 (olive green, diamond); cycle 5 (blue, inverted triangle).

To study the impact of multimodal porosity in pellets for CO_2_ separation kinetics, cyclic adsorption–desorption breakthrough experiments were performed in a mixture of CH_4_ and CO_2_ (55/45 vol%). The breakthrough curves are shown in Fig. 6, where *y*-axis represents the relative concentration of CO_2_ (*C*/*C*_0_) with *C* as the output concentration and *C*_0_ as the initial concentration. Both structured pellets show a sharp breakthrough front, indicating even gas flows within the column and low mass transfer resistance signifying rapid uptake kinetics. In Fig. 6a, the Cu-MOF pellets showed a 7.5% of reduction in CO_2_ uptake after the first breakthrough cycle with the CO_2_ adsorption capacity of 2.6 mmol g^−1^ for the final cycle. This loss of capacity is due to the chemisorbed CO_2_, which could not be removed during the regeneration step. This signifies that more energy is required in the regeneration process, which correlates with the high heat of adsorption value for the Cu-MOF, as discussed earlier. In contrast, the hierarchical Cu-MOF revealed repeatable cycles with a high CO_2_ capacity of 3.1 mmol g^−1^ (Fig. 6b) without any significant reduction in the breakthrough point, indicating that the CO_2_ uptake was primarily due to physisorption and the contribution from the chemisorbed CO_2_ is negligible. Furthermore, the slope of the hierarchical porous Cu-MOF's breakthrough curve is steeper than the slope for the Cu-MOF, indicating a faster mass transfer.

To estimate the mass transfer kinetic parameters, the breakthrough data was simulated using eqn (1) from Klinkenberg, which gives the relative concentration *C*/*C*_0_, at the end of the PSA column:^39^1
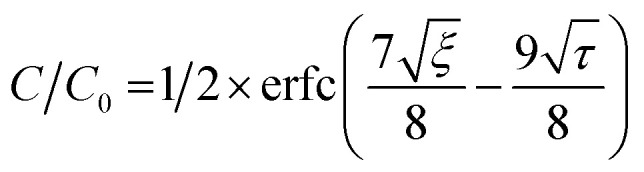
In the above equation, *ξ* and *τ* are dimensionless length (eqn (2)) and dimensionless time (eqn (3)):2
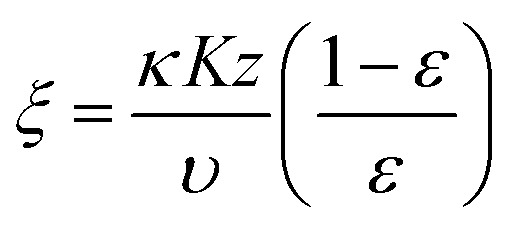
3
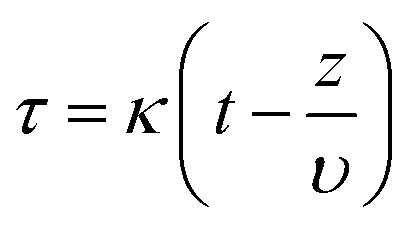
where *k* represents the mass transfer coefficient, *K* is the Henry's constant of the material, *ε* is the void fraction in the PSA bed, *z* is the PSA bed length. Interstitial velocity *υ* is expressed in eqn (4), where *Q*_V_ represents the volumetric gas flow rate and the cross-sectional surface area of the bed is given by *S*.

4 *υ* = *Q*_V_*ε*/*S*

The breakthrough data was fitted according to the eqn (1) to evaluate the mass transfer kinetics. The simulated mass transfer coefficients were 1.2 m s^−1^ and 1.8 m s^−1^ for Cu-MOF and hierarchical Cu-MOF, respectively. This 50% increase in the mass transfer coefficient, signifies high mass transfer rate for CO_2_ from the bulk to the adsorption site owing to the additional mesoporosity. To ensure the hierarchical Cu-MOF pellets efficiency in the CO_2_ separation application, the kinetic performance was compared with the benchmark zeolites from the literature. The mass transfer coefficient of hierarchical Cu-MOF pellets was 38% higher than the benchmark NaX zeolite granules reported earlier.^33^ In summary, this study has shown that shaping nanocrystalline MOF materials into hierarchical porous sorbent structures is an important path to utilize these materials in CO_2_ separation by maintaining high CO_2_ uptake and selectivity and improving mass transfer. In general, creating a structured sorbent with hierarchical porosity is important for efficient gas separation and catalytic processes by tailoring the mass and heat transfer kinetics. Indeed, we show that creating additional porosities at various length scales enhances the mass transfer kinetics in a gas adsorption process and offers the benefit of utilizing a packed column of structured sorbent. Therefore, the present research provides guidelines for designing the hierarchical sorbents for CO_2_ capture. Furthermore, the methodology is facile and can be modified to achieve the desired combination of hierarchical porosities, at various length scales, for a specific gas separation and/or a catalytic process for technological development.

## 4 Conclusion

Here we report the synthesis and nanostructuring of a Cu-MOF, resulting in micro–meso and macroporosity within a single adsorbent. Nanocrystals were synthesized using solvothermal synthesis and were optimized to develop hierarchical Cu-MOF nanocrystals of 150–450 nm with high BET surface area, 627.4 m^2^ g^−1^ with average mesopore size of 27 Å. The hierarchical micro–mesoporous Cu-MOF exhibited a 46% higher CO_2_ adsorption capacity at 1 bar pressure compared to the Cu-MOF and a CO_2_-over-N_2_ selectivity of 12. Additionally, the hierarchical Cu-MOF nanocrystals showed a low isosteric heat of adsorption of only 28 kJ mol^−1^. Compared to most zeolites, this implies a lower energy footprint in a regeneration process. To incorporate pores of all length scales, nanocrystals were structured into pellets to induce additional macropore channels for effective mass transfer. The hierarchical Cu-MOF pellets displayed a high mechanical strength of 1.4 MPa and a stable structure during high-pressure gravimetric CO_2_ adsorption with a capacity of 160 mg g^−1^ at 295 K and 10 bar. The CO_2_ adsorption uptake for the hierarchical MOF is moderate compared to some state of the art MOF like HKUST, MIL-101 and IRMOF-3.^40^ However, the possibility of synthesizing mixed porosity within the structure improves the adsorption capacity of this specific MOF and it results in higher CO_2_/N_2_ selectivity. Furthermore, the breakthrough curve of hierarchical Cu-MOF showed stable cycles with sharp front, implying to low mass transfer resistance with a CO_2_ uptake capacity of 3.1 mmol g^−1^. The mass transfer study showed that hierarchical Cu-MOF exhibit high mass transfer coefficient of 1.8 m s^−1^, greater than the benchmark NaX and CaA freeze-dried zeolite granules reported earlier.^33^ Thus, we believe such a simple strategy to introduce multimodal porosity provides the opportunity to develop high performing nanoscale MOFs for industrial CO_2_ gas separation.

## Author contributions

Kritika Narang Landström: synthesis, experiments, and writing – original draft preparation. Ashwin Nambi: TEM characterization and writing – editing the draft, reviewing, and final draft preparation. Andreas Kaiser and Farid Akhtar – conceptualization, supervision, and writing – editing and reviewing.

## Conflicts of interest

There are no conflicts to declare.

## Supplementary Material

RA-013-D3RA01175E-s001
