# Supplementary material for: Inducing hierarchical pores in nano-MOFs for efficient gas separation
Source: RSC Adv. 2023 May 30;13(24):16039–46. doi: 10.1039/d3ra01175e (PMC10227844; doi:10.1039/d3ra01175e)
Supplement: RA-013-D3RA01175E-s001 [file RA-013-D3RA01175E-s001.pdf]

Electronic Supplementary Material (ESI) for RSC Advances.
This journal is © The Royal Society of Chemistry 2023

Supplementary information

Inducing hierarchical pores in nano-MOFs for efficient gas separation

Kritika Narang Landström[1], Ashwin Nambi[2], Andreas Kaiser[2] and Farid Akhtar [1,*]

[1] Division of Materials Science, Luleå University of Technology, Luleå 97187, Sweden
[2] Department of Energy Conversion and Storage, Technical University of Denmark, 2800 Kgs. Lyngby, Denmark
* Correspondence: farid.akhtar@ltu.se

Additional MOF synthesis protocols:

Table 1S: Protocols of Cu-MOFs synthesized in this work.

Protocol/ Composition	*Metal salt: H_2ADC:DABCO (molar ratio)*	*Solvent (ml)*	*Temperature (℃) and duration (h)*	*BET surface area (m^2/g)*
A(Cu-MOF)	1:1:0.8	27	150 and 48	444
B	2:1:0.5	27	120 and 72	374
C	1:1:0.8	27	120 and 72	537
D(Hierarchical Cu-MOF)	1:1:0.8	90	120 and 48	607
E(Hierarchical Cu-MOF)	1:1:0.8	70	120 and 48	627.4

N_2 adsorption Isotherms

N_2 adsorption isotherms were conducted for all the compositions at 77 K and are displayed in Figure 1S. The N_2 adsorption isotherm of composition E is given in the main article.

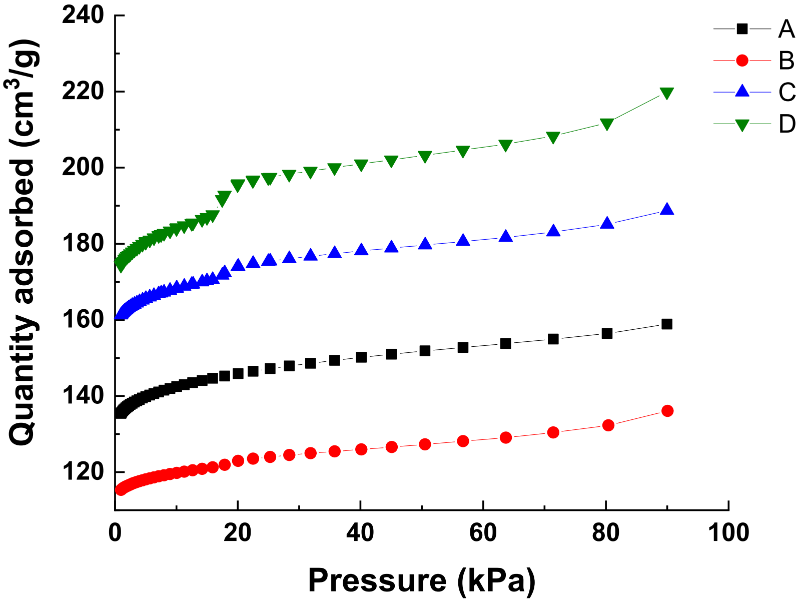

Figure 1S: N_2 adsorption isotherms for composition A (square, black), B(circle, red), C(triangle, blue) and D (inverted, olive green).

Thermal stability

Thermogravimetric analysis of Cu-MOF and hierarchical-Cu-MOF is shown in Figure 2S.

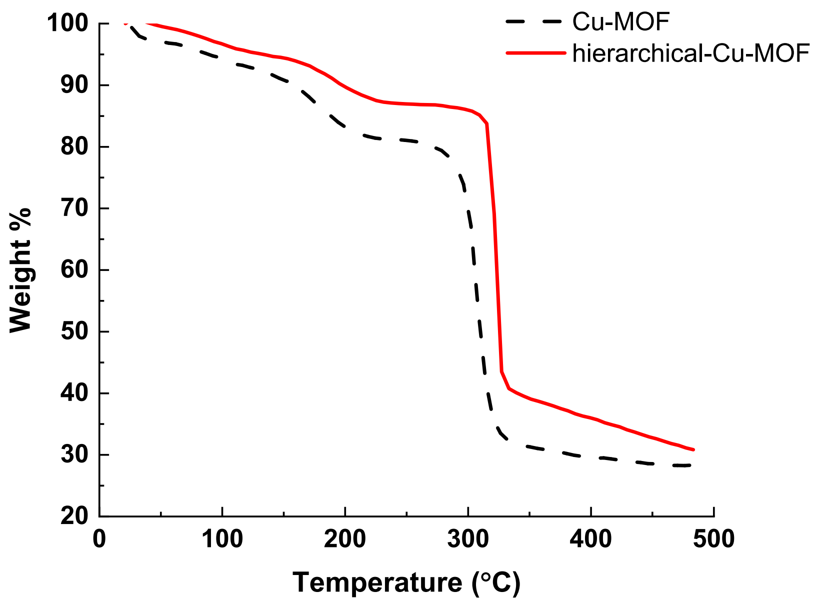

Figure 2S: Thermal gravimetric analysis of Cu-MOF, composition A (black, dashed line) and the hierarchical-Cu-MOF, composition E (red, solid line)

MOF structure

The CIF file of activated $Zn_2(adc)_2(dabco)$ MOF was obtained from the crystallographic database. The structure is simulated using Vesta software and is illustrated in Figure 3S.

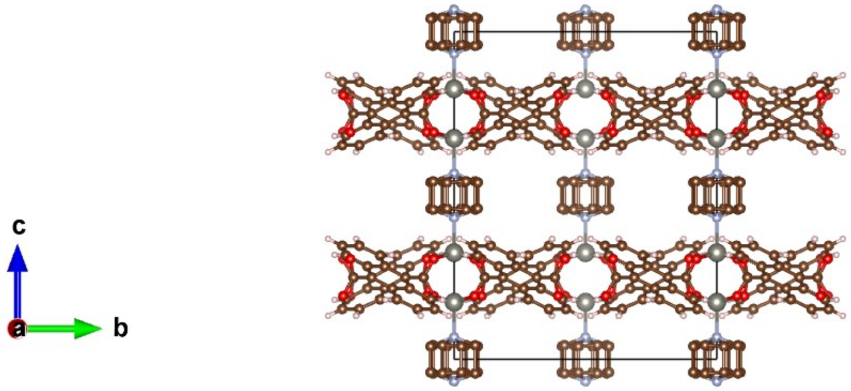

Figure 3S: Structure of $Zn_2(adc)_2(dabco)$ MOF

Table 2S: Selectivities for the separation of CO_2 from CO2/N2 and CO2/CH4 mixtures for hierarchical Cu-MOF and state-of-the art MOFs fro-m literature

MOF Name	***BET surface area (m^2/g)***	***Gas Mixture***	***Selectivity of CO2***	***CO_2 adsorption capacity***	***Tem-perat ure***	***Pressure***	***Refer ence***
Hierarchical Cu - MOF	627.4	CO_2/N_2<br>CO_2/CH_4	12<br>3	75 mg/g	295 K	1 bar	This work
IRMOF-3	2350	CO_2/N_2	18	54 mg/g	298 K	1 bar	[1]
HKUST-1	2211<br>1948	CO_2/CH_4<br>CO_2/N_2	9<br>36	352.1 mg/g<br>484 mg/g	303 K<br>273K	10 bar<br>1 bar	[2]<br>[3]
$Cu_2(Hbtb)_2$	600	CO_2/CH_4	12.4	66 mg/g	298 K	1 bar	[4]
ZnDABCO	1725	CO_2/N_2	17	440 mg/g	298 K	15 bar	[5]
MIL-101	1007	CO_2/CH_4	6	269 mg/g	298 K	10 bar	[6]
UiO-66	1123	CO_2/N_2<br>CO_2/CH_4	61.5<br>18.3	52 mg/g	308 K	2 bar	[7]

References:

1. Y. R. Lee, S. M. Cho, W. S. Ahn, C. H. Lee, K. H. Lee and W. S. Cho, *Microporous Mesoporous Mater.*, 2015, **213**, 161–168.
2. L. Hamon, JolimaîtreE. and G. D. Pirngruber, *Ind. Eng. Chem. Res.*, 2010, **49**, 7497–7503.
3. X. Yan, S. Komarneni, Z. Zhang and Z. Yan, *Microporous and Mesoporous Mater.*, 2014, **183**, 69–73.
4. B. Mu, F. Li and K. S. Walton, *Chem. Comm.*, 2009, 2493.
5. Z. Liang, M. Marshall and A. L. Chaffee, *Microporous and Mesoporous Mater.*, 2010, **132**, 305–310.
6. M. Babaei, S. Salehi, M. Anbia and M. Kazemipour, *J. Chem. Eng. Data*, 2018, **63**, 1657–1662.
7. G. E. Cmarik, M. Kim, S. M. Cohen and K. S. Walton, *Langmuir*, 2012, **28**, 15606–15613.
